# Meta-Analysis of Sleep Deprivation Effects on Patients With Depression

**DOI:** 10.3389/fpsyt.2021.783091

**Published:** 2021-11-30

**Authors:** Baiqi Hu, Chunyan Liu, Tingting Mou, Fangyi Luo, Tingting Lv, Chao Qian, Jian Zhang, Mengfei Ye, Zheng Liu

**Affiliations:** ^1^Department of Psychiatry, Shaoxing Seventh People's Hospital, Affiliated Mental Health Center, Medical College of Shaoxing University, Shaoxing, China; ^2^Department of Behavioral Neurosciences, Science Research Center of Medical School, Shaoxing University, Shaoxing, China; ^3^Department of Neurology, Shaoxing Hospital, China Medical University, Shaoxing, China; ^4^Department of Orthopedics, Shaoxing People's Hospital, The First Affiliated Hospital of Shaoxing University, Shaoxing, China

**Keywords:** sleep deprivation (SD), depression, meta-analysis, patients, review

## Abstract

**Objective:** Depression is a common disorder with a high recurrence rate. Since the effect of sleep deprivation on depression in existing studies were inconsistent, the present study aimed to reassess the effects of SD on patients by performing a meta-analysis of updated research.

**Methods:** PubMed, Embase, the Cochrane Library, and Web of Science were searched for articles before January 20th, 2021. Data on participant characteristics, SD characteristics, adjunctive method and tests for depression were extracted. A comprehensive analysis was conducted to assess the effect of SD on depression and subgroup analysis was used to determine the sources of heterogeneity.

**Results:** In total, 8 articles were included. An SD time of <7 days slightly worsened depression levels [0.24 (−0.21, 0.69); *I*^2^ = 0%; *P* = 0.43], a time of 7–14 days had antidepressant effects [−1.52 (−2.07, −0.97); *I*^2^ = 19.6%; *P* = 0.288], and a time of more than 14 days also worsened depression [0.76 (0.12, 1.40); *I*^2^ = 43.7%; *P* = 0.169].

**Conclusion:** SD may serve as an effective antidepressant measure in humans when the time was 7–14 days, while a time of <7 days and more than 14 days worsened depression.

## Introduction

Depression is a common, debilitating, and potentially lethal disorder that can affect people of all ages (1). More than 300 million people worldwide suffer from depression. The World Health Organization (WHO) ranks depression as the single largest contributor to global disability, accounting for “years of life lived with a disability” in 13.4% of women and 8.3% in men (2, 3). In its most severe form, depression can lead to suicide. Nearly 800,000 people die by suicide each year, and it is the second leading cause of death in the 15–29 year-old age group (4).

Most people with depression have tried at least one antidepressant medication, but medication effects do slowly manifest, and side effects such as insomnia and anxiety can lead patients to try different medications or refuse medication altogether (5, 6). Furthermore, 30–40% of patients are resistant to available antidepressant medications commonly prescribed for major depressive disorder (7).

As a result of difficulties encountered when treating depression, there is an urgent need to find non-pharmacologic therapies. In clinical practice, many non-pharmacologic therapies have attracted special attention, such as sleep deprivation (SD) (5), bright light therapy (BLT) (8), cognitive behavioral treatment (CBT) (9), and repetitive transcranial magnetic stimulation (rTMS) (5). Among these, SD therapy may have rapid antidepressant effects (10). Some clinical studies have shown that SD is an effective treatment for patients with depression (11, 12). Total sleep deprivation (TSD) for one entire night has been found to improve depression symptoms in 40–60% of patients (13). Unfortunately, the therapeutic effects of SD are transient, and depressive symptoms can even return after a subsequent full night of sleep (5, 14).

Some results have indicated that patients who use a combination of antidepressants and SD have a significantly lower tendency to relapse after a full night of sleep than those who do not use this method (15). Many researchers have attempted combining antidepressants with SD, BLT, or CBT to form integrated antidepressant treatments, which have been shown to have positive effects (16, 17). Therefore, it was hypothesized that certain combinations of depression therapy can enhance the therapeutic effects of SD.

Despite a recent meta-analysis (5), comprehensive aggregated data are lacking. The literature on SD lacks randomized controlled trials and has shown inconsistent results. The literatures included by Boland et al. (5) were published relatively early and new relevant articles may have been published in recent years. Michael Ioannou et al. reported a meta-analysis including randomized controlled trials, cohort studies, and case series, which led to low Evidence-based Medicine Grade (18). In that particular article, SD was not the singular variable in the intervention, and the primary outcomes were depressive symptoms, quality of sleep, health-related quality of life, and so on. Thus, too many variables may lead to greater heterogeneity. This article, by performing a meta-analysis, included RCT articles to examine and update the effect of SD on depressed patients by assessing the change in depression scores before and after SD.

The present study aimed to explore the effectiveness of SD for depression levels. The antidepressant effects of SD are primarily reported in humans, yet the time of sleep deprivation has not been standardized across studies, which may yield inconsistent results. Thus, this study explored whether SD treatment for patients with depression requires a more specific treatment course.

## Methods

### Literature Search Strategy

Studies related to the effects of SD on depression in patients were identified by searching four electronic databases, i.e., PubMed, Embase, the Cochrane Library, and Web of Science. The databases were searched for articles before January 20th, 2021. Keywords included the following: (“sleep deprivation” OR “sleep curtailment” OR “sleep restriction” OR “sleep loss”) AND (“depress^*^”) for patients in the title or abstract.

Six authors in pairs, removed duplicates and screening the records, ensuring that all records were independently evaluated by two authors. Mesh in PubMed and EMTREE in Embase were used, and a secondary or supplementary search was then performed. In total, 10,873 records meeting both search criteria were obtained. Articles were screened in duplicate and excluded by keyword (case reports, reviews, and meta-analyses). Then, studies were selected for inclusion or exclusion according to the title and summary. Additionally, relevant original studies cited in the selected articles were also eligible for inclusion. Final inclusion was determined by reading the full text of the studies.

### Inclusion Criteria

All studies included in this article met the criteria described by the participants, intervention, comparison, outcome, and study design (PICOS) according to recommendations by Preferred Reporting Items for Systematic Reviews and Meta-Analyses (PRISMA).

Participants: Human patients were aged between 12 and 80 years and had been diagnosed with depression based on the Diagnostic and Statistical Manual of Mental Disorders (DSM) and International Classification of Diseases (ICD) criteria, regardless of depression type (e.g., bipolar or unipolar) and gender. In addition, patients with serious organic diseases or mental and somatic comorbidities as well as pregnant women were excluded. Intervention: Sleep deprivation as an intervention. Comparison: The only difference between the experimental group and the control group was that the experimental group had sleep deprivation while the control group did not. In a word, except for sleep deprivation, the rest of the interventions were the same in both groups. As long as such an experimental design was met, we could include the article regardless of how many combination therapies it had. Outcomes: Outcomes were depression assessment tools, such as the HAMD, Beck Depression Inventory (BDI), and Montgomery-Asberg Depression Rating Scale (MADRS). Study design: All included literature were randomized controlled trials.

Articles lacking either the full text or primary data findings that could not be resolved with the Engauge Digitizer tool were excluded.

### Data Extraction and Quality Assessment

Each article was read in its entirety by two researchers to extract the data and record trial details in a standardized table containing the following information: author(s), year of publication, country, participant characteristics (e.g., sample size, age, gender, and sample type), SD characteristics (e.g., type and timetime), adjunctive method (e.g., bright light therapy, cognitive behavioral treatment, and antidepressant drug), and patient outcome. Since the time of a single sleep deprivation and the frequency of sleep deprivation varied in the included articles, we used the concept of total sleep deprivation time including the total numbers of wake nights and recovery time. The time points for the assessment of depressive conditions were the baseline and after the total sleep deprivation intervention.

When no specific data were included (e.g., only graphs or figures), the authors were contacted and asked to provide the results of the experiments or the raw data. If this failed, data were estimated based on graphs or figures using a digital ruler called the Engauge Digitizer (19, 20). Primary data were estimated according to coordinate positions, and then statistical methods were used to calculate the mean and SD. The risk of bias was estimated independently by two researchers (J. Y. and T. M.) who extracted and appraised the data. They used the Cochrane Risk of Bias tool for patient studies (21). Inconsistencies between the two researchers were resolved through negotiation, and when this failed, a third professional was asked to judge the risk of bias.

### Data Synthesis and Analysis

First, to assess the total effects of SD on depression, the selected trials were analyzed without considering characteristics and variables. Then, hierarchical analysis based on a significant variable (in this case, the time of SD) and its effects on patients was performed. Subsequently, subgroup analysis was used to determine the sources of heterogeneity. Subgroup analysis was performed according to the country and adjunctive method for the human studies. For each comparison, standardized mean differences based on Hedge's g were calculated as measures of the effect size, with values ranging from small (0.2–0.5), medium (0.5–0.8), and large (0.8 and higher), as per standard convention. This approach ignored differences in depression measurement tools so that the analysis was unified. The random effects model proposed by DerSimonian and Laird (22) was also used. Finally, publication bias was evaluated by funnel plots and Egger's test to quantify the bias.

The heterogeneity of effect size within each comparison was tested using Cochran's Q test and *I*^2^ statistics. *P*-values of < 0.05 indicated high heterogeneity. Data were presented as the effect size ± confidence intervals at 95%. Results were considered significant when the confidence interval range was lower or higher than zero. All calculations were performed using Stata software (version 13.1).

## Results

### Study Characteristics

**Figure 1:**
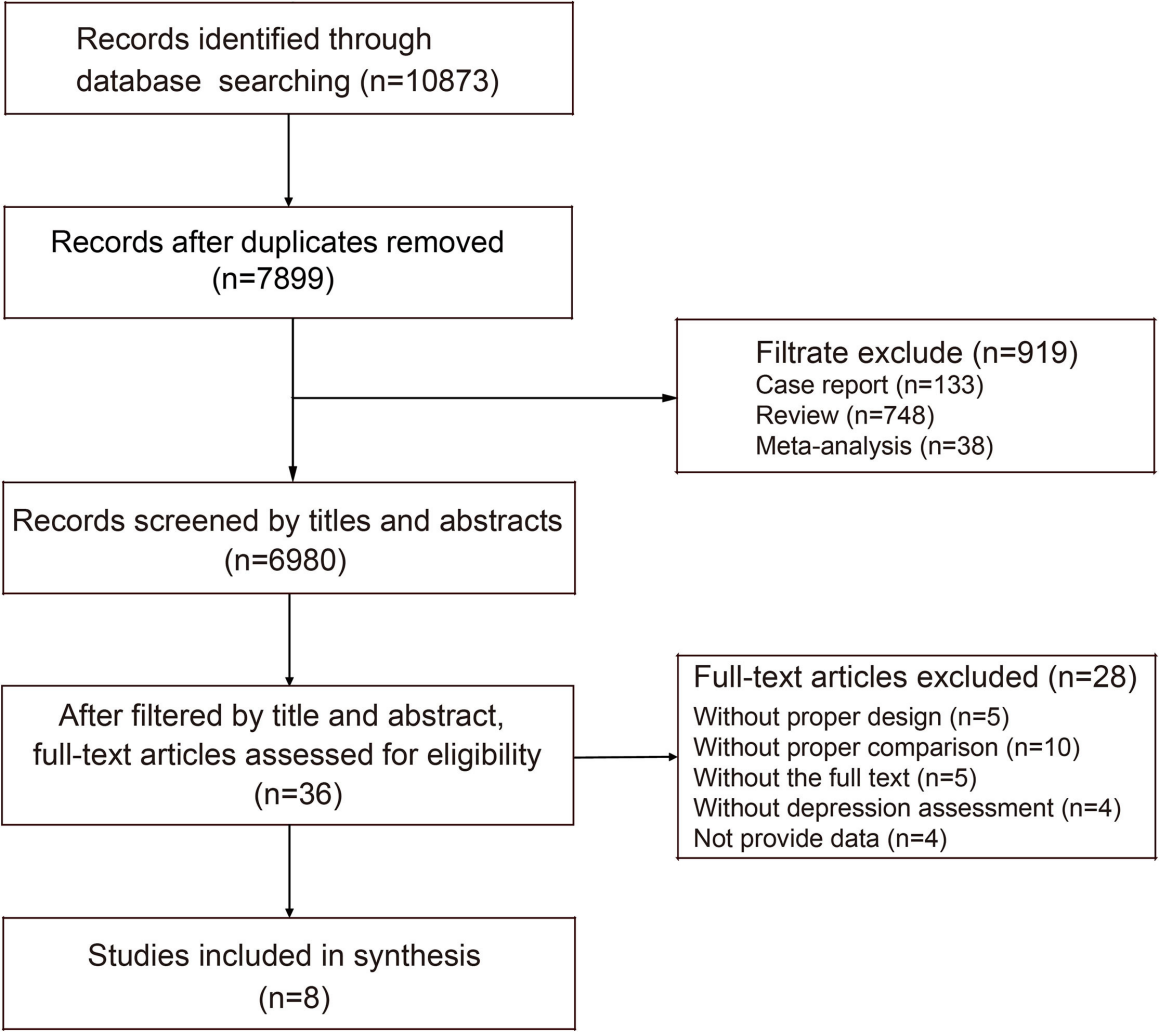
Selection process for trials included in the meta-analyses.

The search strategy resulted in 10,873 articles from PubMed and other databases (Figure 1). Redundant literature was eliminated, and articles were further screened for relevance according to keywords (e.g., case report, review, and meta-analysis). After these steps, a total of 6,980 articles on humans were analyzed according to the title and summary. After analysis of titles and abstracts, 36 articles remained and were screened by reading the full text. After excluding articles that lacked a control group or primary data, a total of eight studies (23–30) involving nine trials meeting the inclusion criteria were ultimately included in the present meta-analysis.

In the studies, TSD was applied in five articles, while partial sleep deprivation (PSD) was applied in three articles. No record of sleep curtailment, sleep restriction, or sleep loss was included. The included studies were conducted in Germany, the United States of America (USA), Turkey, Switzerland, and the Netherlands. All studies involved a combination of SD and other interventions. For instance, six involved antidepressant drugs, and the other two involved either BLT or CBT (Table 1).

**Table 1:** A table of the characteristics of the included studies.

**Author(s)**	**Country**	**Intervention**	**Sample size**	**Age (mean, year)**	**Type of depression**	**SM**	**Combined with other interventions**	**Time**	**Depression scale**
Elsenga et al. (25)	Netherlands	Clomipramine/SD	10	49.1 ± 13.6	Unipolar	TSD	Clomipramine	7 days	Hamilton interview ratings
		Clomipramine	10	55.6 ± 13.2					
Holsboer-Trachsler et al. (30)	Switzerland	Trimipramine/SD	14	50.43 ± 7.3	Bipolar	PSD	Trimipramine	28 days	HRS, MADRS
		Trimipramine	14	50.64 ± 8.50					
Kuhs et al. (23)	Germany	Amitriptyline/LSD	27	43.3 ± 13.6	Bipolar	LPSD	Amitriptyline	14 days	HAM-D, 10 Item
		Amitriptyline	24	46.0 ± 11.3					
Caliyurt et al. (27)	Turkey	LPSD/Sertraline	13	38.46 ± 12.03	Unipolar	LPSD	Sertraline	14 days	HAM-D, 21 Item
		Sertraline	11						
Kundermann et al. (26)	Germany	TSD+CBT	9	37 ± 2.7	Unipolar	TSD	CBT	21 days	HDRS
		CBT	10	37.4 ± 2.6					
Gorgulu et al. (28)	Turkey	TSD/Sertraline	19	40 ± 11.69	Unipolar	TSD	Sertraline	7 days	HAM-D
		Sertraline	22	33.27 ± 11.18					
Smith et al. (29)	USA	TSD/Paroxetine	7	69.0 ± 4.6	Unipolar	TSD	Paroxetine	36 h	HDS, 13 Item
		TSD/Placebo	6	68.6 ± 4.9					
		Paroxetine	3	71.4 ± 6.0					
Gest et al. (24)	Germany	Wake/BLT	25	16.2 ± 1.3	Unipolar	TSD	BLT	One night	BDI-II
		BLT	37	15.8 ± 1					

*BDI-II, Beck Depression Inventory-II; BLT, Bright Light Therapy; CBT, Cognitive behavioral therapy; HAM-D, Hamilton Depression Scale; HAM-D, 10 Item, Hamilton Depression Scale (10-item); HAM-D, 21 Item, Hamilton Depression Scale (21-item); HDRS, Hamilton Depression Rating Scale; HDS-13, Hamilton Depression Scale (13-item); HRS, Hamilton Rating Scale for Depression; LPSD, late partial sleep deprivation; LPSD, late partial sleep deprivation; MADRS, Montgomery–Asberg Rating Scale; PSD, partial sleep deprivation; SD, sleep deprivation; SM, sleep manipulation; TSD, total sleep deprivation; USA, United States of America*.

### Study Quality

**Figure 2:**
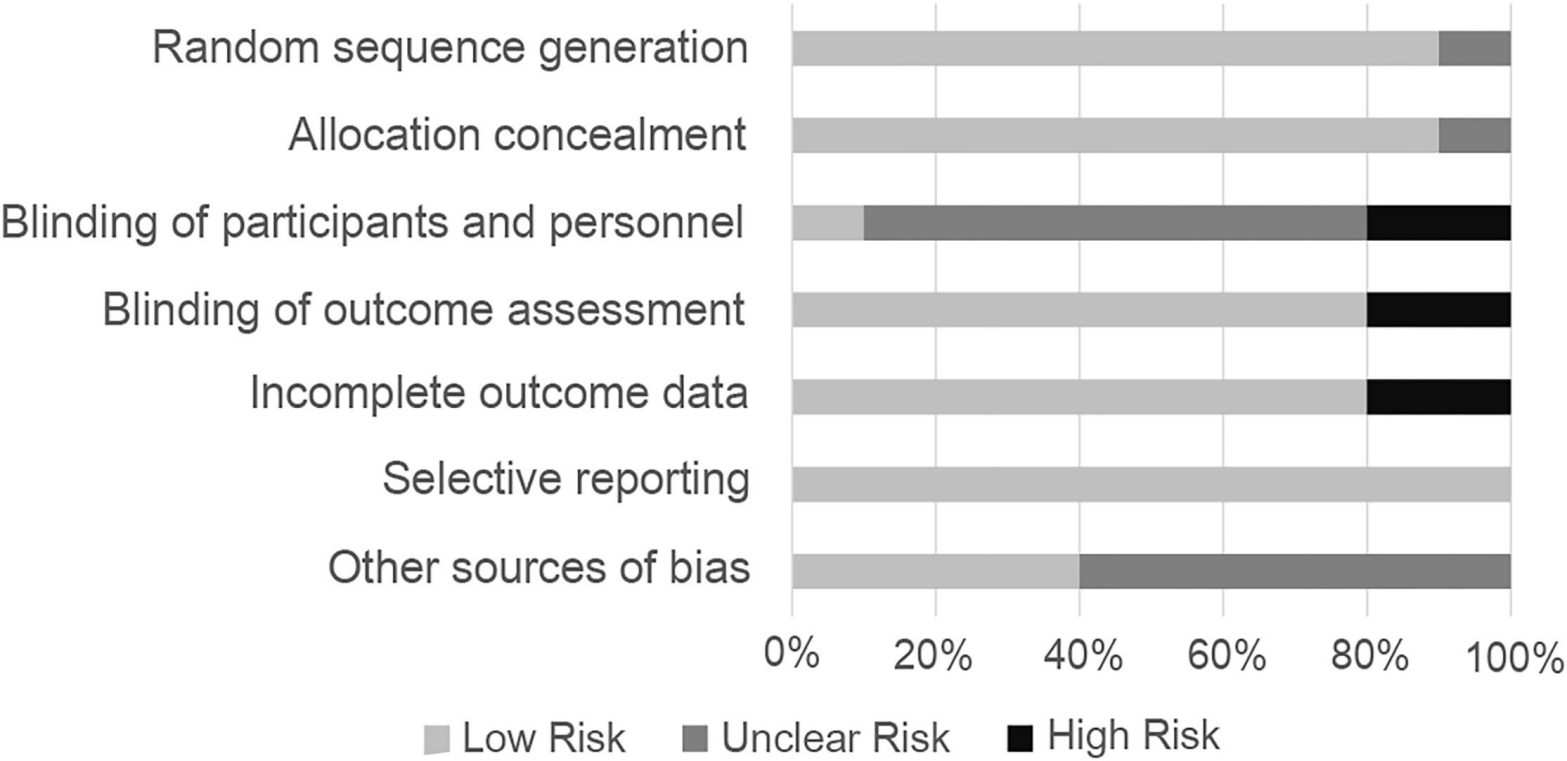
Risk-of-bias assessments of the included studies.

**Figure 3:**
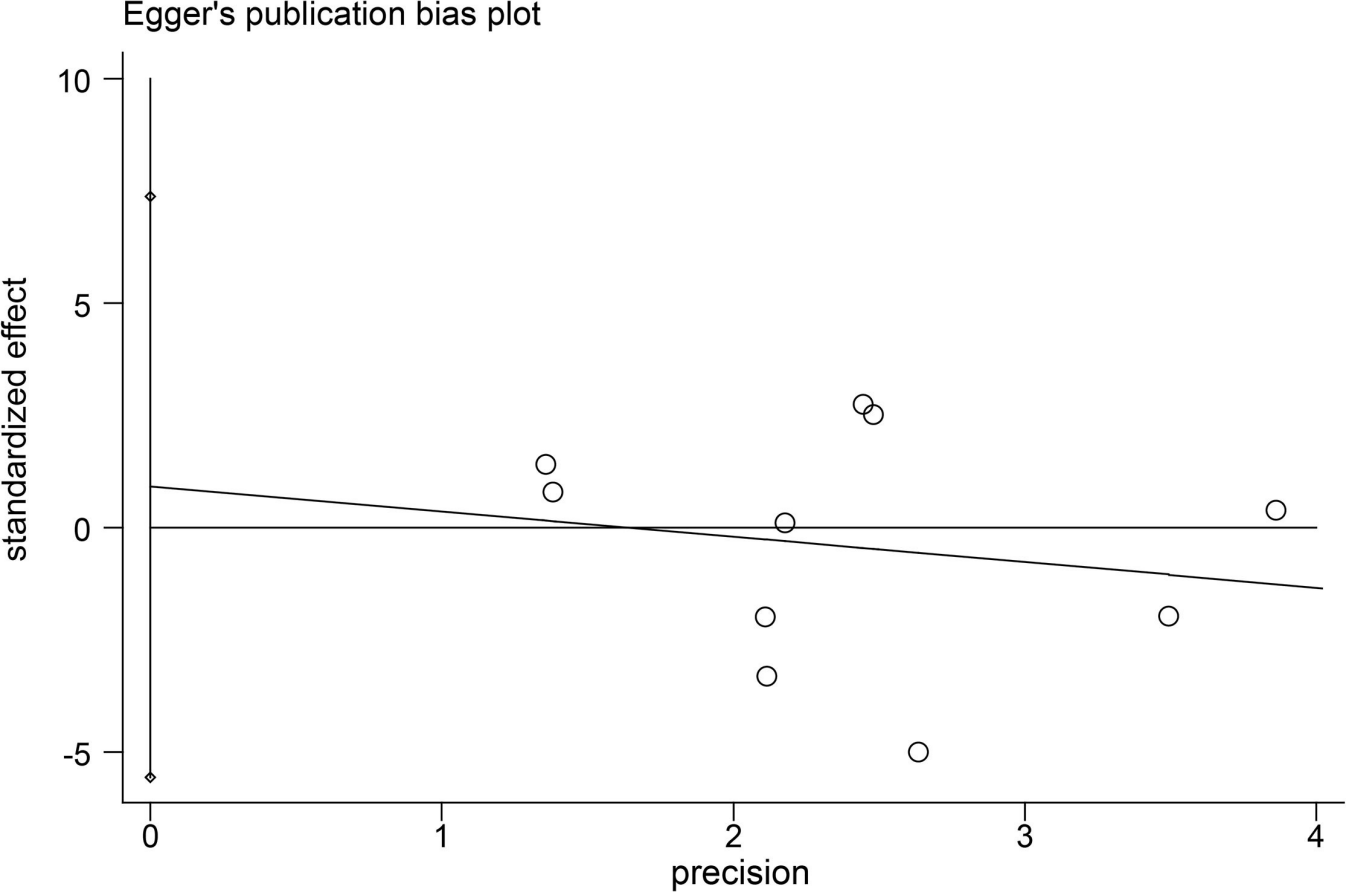
Egger's test.

Most studies adopted a randomized control trial (RCT) method, in which most random sequence generations indicated a low risk of bias (22). Performance bias was not mentioned in most of the articles and was therefore a mostly unclear bias risk. Although the articles did not mention detection bias, the degree of depression was quantitatively measured by the depression scale; therefore, the tested factor had little influence, and the authors believed there was a low risk of detection bias. Two studies did not blind participants (27, 28), and other articles did not mention blindness. However, SD was not possible to blind a human being exposed to, which was considered a shortcoming of those studies. The final data for one study were unclear; thus, a high risk of bias was identified for the outcome of that study (30). Unclear bias accounted for the majority of other biases, since some literature only provided images instead of concrete data; thus, the data obtained through software processing may have had some impact on the results (Figure 2). Besides, egger's tests showed that there were no indications of publication bias (Figure 3).

### Main Efficacy of the Meta-Analysis

**Figure 4:**
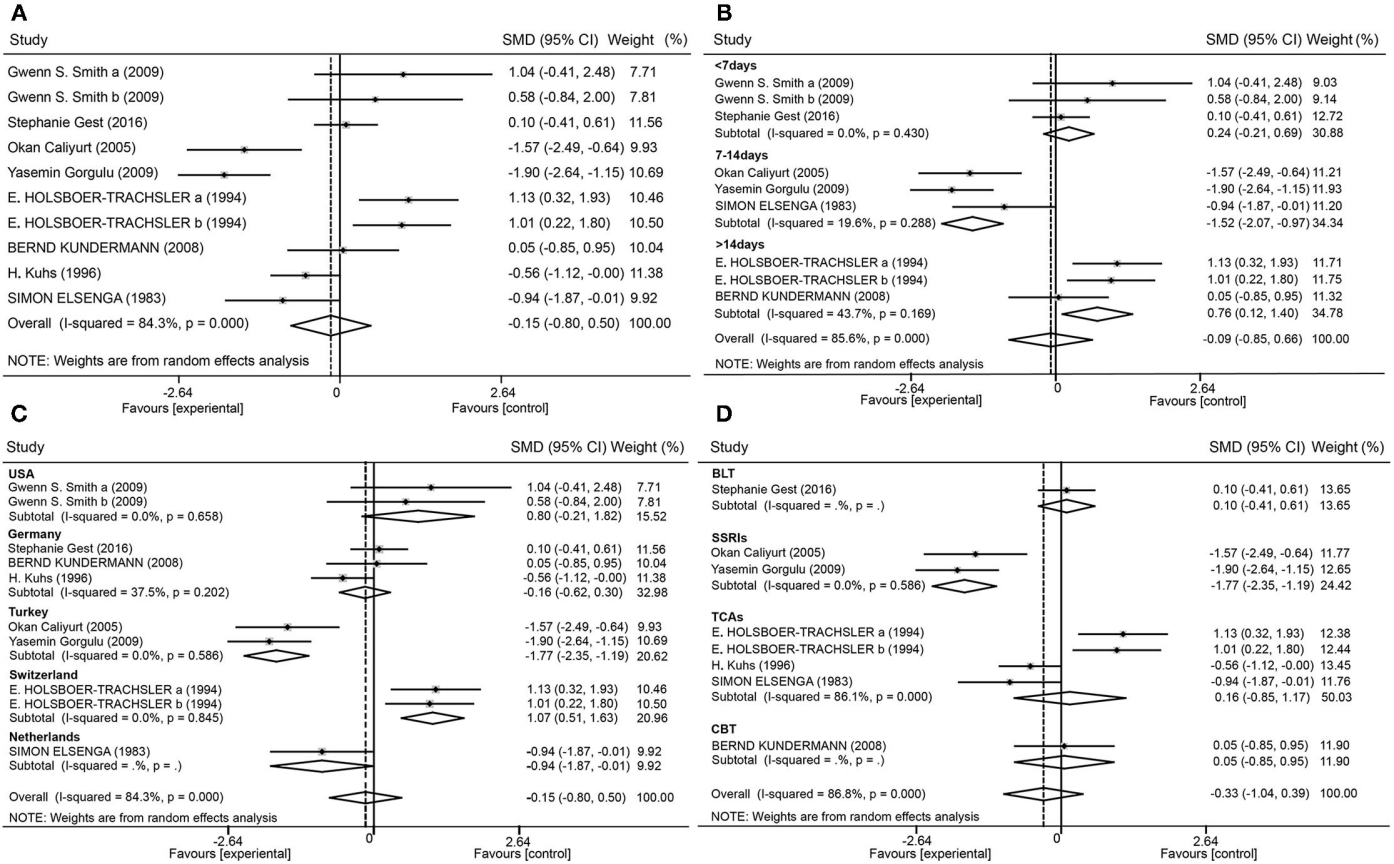
Forest plot. Forest plot of the random effects model meta-analysis of SD effects **(A)**; and subgroup analysis of patients with regard to the time of SD **(B)**, the country **(C)**, and combined therapy **(D)**.

Figure 4 shows the total effect of SD on depression. Nine trials (10 datasets) reported depression using the HAMD. The random effects meta-analysis elicited a summary effect size of −0.15 (95% confidence interval [CI], −0.80 to 0.50; *I*^2^ = 84.3%; *P* = 0.000) (Figure 4A). When analyzed according to the SD schedule (<7 days, 7–14 days, or >14 days), the forest plot showed that an SD time of <7 days slightly worsened depression levels [0.24 (−0.21, 0.69); *I*^2^ = 0%; *P* = 0.43]. Finally, a time of 7–14 days had significant antidepressant effects [−1.52 (−2.07, −0.97); *I*^2^ = 19.6%; *P* = 0.288], and a time of more than 14 days also moderately worsened depression [0.76 (0.12, 1.40); *I*^2^ = 43.7%; *P* = 0.169] (Figure 4B).

### Heterogeneity Analyses

By performing subgroup analysis, sources of research heterogeneity were identified. These may have been related to the country in which the research was conducted, type of combination therapy employed, and depression tests that were used. Studies were divided into five subgroups according to country (Figure 4C). Studies from Turkey showed large antidepressant effect sizes [−1.77 (−2.35, −1.19); *I*^2^ = 0%; *P* = 0.586], while studies from Switzerland showed large effect sizes for worsened depression [1.07 (0.51, 1.63); *I*^2^ = 0%; *P* = 0.845]. The studies were also divided into four subgroups according to the combination of therapies (Figure 4D). Studies that combined selective serotonin reuptake inhibitors (SSRIs) with SD showed antidepressant effects [−1.77 (−2.35, −1.19); *I*^2^ = 0%; *P* = 0.586].

To investigate gender as a variable, a subgroup analysis was performed, and grouping was determined by the proportion of women in the total population. When the ratio was >0.8, the effect size was 0.31 (95% CI, −0.46 to 1.09; *I*^2^ = 30.7%; *P* = 0.230). When the ratio was between 0.5 and 0.8, the effect size was −0.97 (95% CI, −1.69 to −0.26; *I*^2^ = 71.6%; *P* = 0.007). When the ratio was <0.5, the effect size was 0.76 (95% CI, 0.12–1.40; *I*^2^ = 43.7%; *P* = 0.169). After one paper was removed via sensitivity analysis (23), it was revealed that the heterogeneity of the 7–14-day group decreased from 66.6 to 19.6%, indicating that the effects of SD on depression were related to SD time in the studies.

## Discussion

All eight articles in this meta-analysis used RCT models, which helped to improve the rigor and significance of the review. Judging from the total results of the studies, the data were not as obvious as those revealed in previous meta-analyses and were highly heterogeneous (5). This was because new research was included, and digital software was used to address instances of incomplete data.

In the <7 days group, SD only occurred once with a time of <36 h and the results showed that SD slightly worsened depression. Several articles reported that depression symptoms returned immediately after SD and recovery, with some patients experiencing more severe depression than before (14, 15). The included studies in our study assessed depression after SD, therefore, it was likely that a recurrence of depression following short time of SD may be the cause of this outcome. Sleep loss, especially when chronic, can cause significant and cumulative neurobehavioral deficits and physiological changes, which may present as inattention, slowed working memory, reduced cognitive throughput, depressed mood, and perseveration of thought (31). Thus, prolonged and repeated SD may worsen depression, which could account for the increased depression in groups who were subject to an SD time longer than 14 days.

With 7–14 days of SD, interference of the first two conditions was slightly prevented, thus providing a better therapeutic effect. The heterogeneity of this group mainly originated from differences in sample type among the three articles (23). It has been debated whether the polarity of depression affects the response to SD. Studies indicated that in unipolar depressed samples, the response rate to SD was 50.6%, and in samples using a mixture of unipolar and bipolar depressed patients, the response rate was 53.1% (5). And in another meta-analysis, they found similar numerical yet not statistically significant effect sizes for patients with bipolar depression and non-elderly patients with unipolar depression (18). However, with the small amount of literature included in this study, it was impossible to clearly explore similar results. And in the future, it is a question worthy of discussion.

Given the large heterogeneity in the total dataset, sources of heterogeneity were explored for potential influences on variables. The first analysis was a subgroup analysis according to country. Different countries present different factors affecting the occurrence, treatment, and prognosis of depression, such as national health awareness, cultural and quality of education, medical research level, medical and social security, family economic income, social welfare, and social support systems (32, 33). In studies from Turkey, there was an antidepressant effect of SD [−1.77 (−2.35, −1.19); *I*^2^ = 0%; *P* = 0.586]. However, studies from Switzerland showed that SD worsened depression [1.07 (0.51, 1.63); *I*^2^ = 0%; *P* = 0.845]. These findings suggest that the effects of SD on depression may be related to ethnicity and nationality.

Although relatively few articles were included in this study, based on the available data, it was speculated that the treatment effects of SD on depression are more likely to be observed in studies conducted in the Turkish context. An adverse effect of SD was observed in patients from Switzerland in one paper, so additional studies are needed for verification. In light of the above, it should be noted that Turkey has low levels of economic and medical academic development and education, while Switzerland has high levels of the same indices. Depression levels in the Turkish studies may have been significantly related to the medical academic development. In consideration of the possible effects of different ethnic characteristics, further studies are needed to examine the effects of SD on depression across various ethnic groups.

Another subgroup analysis was dependent on whether there was a combination of SD with other therapies. Combination therapy with BLT, CBT, and tricyclic antidepressive agents (TCAs), showed no significant effects of SD on depression. Three studies on SD combined with SSRIs showed antidepressant effects. After removing one study (29) with fewer than seven patients in each group, which may have affected the outcome, the effect size changed from [−0.58 (−1.94, 0.78)] to [−1.77 (−2.35, −1.19)], and heterogeneity changed from 84.3 to 0%.

It was suspected that heterogeneity was related to three aspects. (1) Patients in one of the studies were from the USA, and those in the other two were from Turkey. This correlated with the results of the above analysis, namely that the Turkish studies showed great antidepressant effect of SD and thus an overall effect was antidepressant. (2) One study incorporated paroxetine, while sertraline was used in the other two, so heterogeneity may have originated from the use of different SSRIs. The authors of one paper stated that although sertraline and paroxetine had comparable efficacy for major depression, patients who used sertraline showed lower recurrence rates than those who used paroxetine. Sertraline was somewhat better tolerated than paroxetine and induced lower side-effect profiles (34). (3) Finally, the time of SD in one study was <7 days, while the time was 7–14 days in the other two studies. Furthermore, TCA was not very effective but at the same time, two of the studies were conducted in Switzerland while the TCA studies not from Switzerland did indeed show small positive effects, which was related the above result that studies from Switzerland showed that SD worsened depression. Regarding the gender variable, an effect size between 0.5 and 0.8 was inconsistent with other figures, so it was suggested that gender was not an influential factor in the treatment effects of SD on depression.

In addition, it was worth mentioning that the article by Gest et al. (24) analyzed SD in adolescents. As far, there was very little evidence on chronotherapeutics (such as BLT and SD) in children and adolescents. However, since chronobiology in adolescents differed from that in adults (35) and the evidence for the effects of chronotherapy in children and adolescents were indeed mostly positive but of very low quantity (24, 36), such discrepancies may affect the results and longer-term studies of adolescents are needed. Furthermore, there was no information on sleep phase advance (SPA) in our article, which was also a relevant form of chronotherapy. SPA consists in manipulating the sleep–wake cycle by supporting sleep earlier than the patient's usual bedtime and wakefulness before the usual waking time. D'Agostino et al. (37) suggested that triple chronotherapy (SD-BLT-SPA) might be a safe and effective addition to conventional antidepressant interventions. Although SPA was not mentioned in the studies we included, future studies could try to combine SD, BLT and SPA in the treatment of depression and explore their effects.

The mechanisms of SD regarding depression treatment are complex and are thought to be based on monoaminergic neurotransmission, neuroplasticity, and gene expression. Brain-derived neurotrophic factor (BDNF) levels have been shown to be reduced in individuals suffering from major depressive disorder, and decreased levels are also negatively correlated with Hamilton Rating Scale for Depression (HAM-D) scores. Use of SD has resulted in faster treatment response and increased BDNF levels (28). One study (38) found that in patients who achieved an antidepressant effect after SD, the expression of specific circadian clock genes (e.g., RORA, DEC2, and PER1) increased. However, in patients that did not show this effect, a significant decrease in the expression of these genes was found (39).

Considering that the mechanisms underlying SD-induced or SD-inhibited depression are poorly understood, it is a natural choice to consider animal studies. Animal models are a cornerstone of human research, particularly in research on depression at the tissue, cellular, molecular, and genetic levels. However, no relevant meta-analyses have provided comprehensive results regarding animal studies of depression. Perhaps, we can look forward to animal-related studies that will allow us to compare the effects of sleep deprivation on animals and humans.

## Implications

Based on this study's findings, confining the time of SD treatment to 7–14 days may be a clinically feasible way to enhance its therapeutic effects. Regarding combination treatment, SD in addition to SSRI medications was an option, but the time of SD, different medications and countries needed to be considered. Combined with clinical practice, TCAs yield more troublesome side effects and potentially lead to fatal overdose; thus, SSRIs may be safer (40). This study did not include papers with methods combining three or more therapies, so further investigation is needed.

## Limitations

Aside from the abovementioned speculations, several limitations should be noted. The quantity of literature was small, which made the results less convincing and could also have compromised the power of the funnel plot. Second, the data derived using digital software were different from the actual study results, so there was a certain degree of data error. Third, included studies may differ in concern to SD protocols, such as the time of single SD and frequency of SD, which was typically a relevant issue in chronotherapy research and may then lead to heterogeneity.

## Conclusion

This meta-analysis showed that SD may serve as an effective antidepressant measure in humans when the treatment time was 7–14 days. A time of <7 days slightly worsened depression, while a time of more than 14 days certainly worsened depression. These findings suggest that SD may be used as an intervention for depressed people within specific parameters. Additional high quality research with longer follow-up is needed to strengthen this evidence.

## Data Availability Statement

The original contributions presented in the study are included in the article/supplementary material, further inquiries can be directed to the corresponding authors.

## Author Contributions

BH, MY, and CL wrote the protocol, managed the literature searches, analyzed data, and wrote the draft of the manuscript. ZL designed the study, wrote the protocol, and revised the manuscript. TL, TM, and FL managed the literature searches and analyses. TL undertook the statistical analysis. MY, CQ, and JZ modified the manuscript. All authors contributed to the article and approved the submitted version.

## Funding

This work was funded by the Public Welfare Technology Applied Research Projects in Zhejiang Province (no. 2016C33191) and the Zhejiang Medical Health Science and Technology Project (no. 2019KY724 and 2020KY332).

## Conflict of Interest

The authors declare that the research was conducted in the absence of any commercial or financial relationships that could be construed as a potential conflict of interest.

## Publisher's Note

All claims expressed in this article are solely those of the authors and do not necessarily represent those of their affiliated organizations, or those of the publisher, the editors and the reviewers. Any product that may be evaluated in this article, or claim that may be made by its manufacturer, is not guaranteed or endorsed by the publisher.

## Abbreviations

BDNF: brain-derived neurotrophic factor
BLT: bright light therapy
CBT: cognitive behavioral treatment
HAM-D: Hamilton Rating Scale for Depression
PICOS: participants, intervention, comparison, outcome, and study design
PRISMA: Preferred Reporting Items for Systematic Reviews and Meta-Analyses
PSD: partial sleep deprivation
RCT: randomized controlled trials
rTMS: repetitive transcranial magnetic stimulation
SD: sleep deprivation
SMD: standard mean difference
SSRIs: selective serotonin reuptake inhibitors
SPA: sleep phase advance
TCA: tricyclic antidepressive agent
TSD: total sleep deprivation.
